# Longitudinal associations between problematic Internet use, self-esteem, and depressive symptoms among Chinese adolescents

**DOI:** 10.1192/j.eurpsy.2022.385

**Published:** 2022-09-01

**Authors:** W. Lai, W. Wang, L. Guo, C. Lu

**Affiliations:** Sun Yat-sen University, School of Public Health, Department Of Medical Statistics And Epidemiology, Guangzhou, China

**Keywords:** problematic Internet use, self-esteem, adolescence, depressive symptoms

## Abstract

**Introduction:**

Adolescents spend more time on the Internet than adults, making them susceptible to problematic Internet use (PIU). Evidence shows that PIU has a negative impact on self-esteem among adolescents, disturbing the development of emotional regulation, which makes them more likely to develop depressive symptoms subsequently. However, there is lack of literature focusing on the process that self-esteem may mediate the association between PIU and depressive symptoms.

**Objectives:**

This study aimed to examine the prospective links between PIU, self-esteem, and depressive symptoms in adolescence.

**Methods:**

A total of 1,736 adolescents completed this longitudinal study. The baseline survey was conducted in 2019, and the follow-up surveys were performed at 1-year and 2-year later. Problematic Internet use, self-esteem, and depressive symptoms were measured. A cascade model was used to examine the longitudinal associations between PIU, self-esteem, and depressive symptoms.

**Results:**

The mean (SD) age of participants was 13.6 (1.5) years at baseline. The final results observed significant within-time associations between PIU, self-esteem, and depressive symptoms at each time point. PIU and low level of self-esteem could predict subsequent depressive symptoms among adolescents, and depressive symptoms were also associated with subsequent PIU and self-esteem.

**Conclusions:**

Both problematic Internet use and self-esteem show bidirectional predictions with depressive symptoms among Chinese adolescents. Health-related professionals, schools and families should be aware of the findings of bidirectional associations. Adolescents with problematic Internet use and lower self-esteem should be paid more attention to attenuate the risk of developing depressive symptoms.

**Disclosure:**

No significant relationships.

